# Effects of *Rubus fruticosus* and *Juniperus oxycedrus* derivatives on culturability and viability of *Listeria monocytogenes*

**DOI:** 10.1038/s41598-022-17408-4

**Published:** 2022-08-01

**Authors:** Federica Barbieri, Chiara Montanari, Vida Šimat, Danijela Skroza, Martina Čagalj, Sonja Smole-Možina, Daniela Bassi, Fausto Gardini, Giulia Tabanelli

**Affiliations:** 1 Department of Agricultural and Food Sciences, University of Bologna, Piazza Goidanich 60, 47521 Cesena, Italy; 2 University Department of Marine Studies, University of Split, 21000 Split, Croatia; 3 Department of Food Technology and Biotechnology, Faculty of Chemistry and Technology, University of Split, 21000 Split, Croatia; 4 Department of Food Science and Technology, Biotechnical Faculty, University of Ljubljana, 1000 Ljubljana, Slovenia; 5 Department for Sustainable Food Process (DISTAS), University Cattolica del Sacro Cuore, 26100 Cremona, Italy; 6 Interdepartmental Centre for Industrial Agri‑Food Research, University of Bologna, 47521 Cesena, Italy; 7 Department of Agricultural and Food Sciences, University of Bologna, 40127 Bologna, Italy

**Keywords:** Antimicrobials, Bacterial physiology, Pathogens

## Abstract

The consumers’ demand for safe foods without chemical additives increased the research for green solutions, based on natural antimicrobials. Plants can be an important source of bioactive compounds able to prevent the development of foodborne pathogens and spoilage microflora. This paper aimed to characterize phenolic extracts (PEs) and essential oils (EOs) obtained from Mediterranean *Rubus fruticosus* leaves and *Juniperus oxycedrus* needles and to evaluate their antimicrobial effects against *Listeria monocytogenes* Scott A. The growth dynamics with sub-lethal concentrations of plant derivatives were modeled and flow cytometry was used to better evidence the effect on cell viability and culturability. The results showed that these plant derivatives affected the growth of *L. monocytogenes*, increasing lag phase (about 40 h in the presence of PEs vs. 8 h in the control) and decreasing the final cell load of at least 1 log cycle with respect to the control. *R. fruticosus* EO was the most effective, determining an initial decrease of cell counts of about 6 log cycles, followed by a restart of growth after 10 h, with rate similar to the control (0.08 with *R. fruticosus* EO vs. 0.09 ((log CFU/ml)/h in the control) but significantly lower final cell load (7.33 vs. 8.92 log CFU/ml). According to flow cytometry, only *R. fruticosus* EO induced a relevant increase of dead cells, while the other plant derivatives determined different extent of sub-lethal cell injury. The discrepancy observed in some cases between viability and culturability could indicate the presence of cells not able to grow in culture media, whose fate needs to be further investigated to assess their potential recovery, thus bringing to an overestimation of the antimicrobial effect of these substances. This research contributed to increase the knowledge of these underused raw materials such as blackberry leaves and juniper needles that can be exploited in food and other industries.

The request of consumers for safe foods without chemical additives is addressing the recent research towards the replacement of chemical preservatives with natural molecules that show a broad spectrum of antimicrobial potential^1,2^. In this perspective, plants can be an important source of molecules that possess strong activities against many microorganisms associated with food spoilage or safety. Indeed, derivatives of many aromatic plants have been used for centuries in medicine, cosmesis and food production, independently of the real comprehension of their antimicrobial role. Besides, many of these substances are generally recognized as safe (GRAS), being derived from edible products, and widely accepted by consumers. For this reason, many plant extracts, essential oils (EOs) or their constituents have been proposed as potential preservative ingredients in several foods or as antimicrobial agents^2,3^.

Mediterranean maquis is a natural ecosystem with many aromatic plants carrying these characteristics^4–8^. In this habitat, species of the *Rosaceae* family are extremely widespread. In particular, *Rubus fruticosus,* together with *Rubus ulmifolius*, two plant varieties commonly known as blackberry, are an example herein. Fruits, leaves, and young shoots of these species have been used in traditional medicine for several purposes^9^. Their phenolic extracts (PE) exhibit relevant antioxidant activity^10^, being rich in phenolic acids, flavonoids (anthocyanins, flavonols and tannins), carotenoids and organic acids^11^. Many of the molecules responsible for the antioxidant activity can inhibit the growth of microbial species. Veličković et al.^11^ found promising antimicrobial activity (especially against *Listeria monocytogenes*) in different aqueous and acetone extracts of *Rubus discolor*. Bioactive phenolics from blackberry pomace have been proposed to reduce *Salmonella* contamination in farm animals^12,13^. *R. fruticosus* showed relevant antimicrobial activity against several microorganisms, including the bacteria *Escherichia coli*, *Salmonella* Typhi, *Staphylococcus aureus*, while none or scarce effects were observed against yeasts and moulds, as revealed by the minimum inhibitory concentrations (MIC) reported^14^. The blackberry juice was characterized by the ability to reduce the growth of *L. monocytogenes, E. coli* O157:H7, and *Salmonella* Typhimurium, while the same juice significantly stimulated the growth of the lactic acid bacteria *Lactiplantibacillus plantarum*, *Lacticaseibacillus casei* and *Lacticaseibacillus rhamnosus*^15^.

Another genus of the Mediterranean maquis is *Juniperus*, belonging to the Cupressaceae family. To this genus, belong about 70 species. Among the most widespread species, there are *Juniperus communis, Juniperus turbinata, Juniperus deltoides* and *Juniperus oxycedrus*. *Juniperus oxycedrus* has, for example, traditional history of use in therapeutic and folk medicine for different diseases, such as tuberculosis, pneumonia, bronchitis, diarrhea, stomach aches, and hyperglycemia, but the data available on the chemical composition and biological activity are scarce^16,17^. Extracts of juniper species have been widely studied in relation to antioxidant activity^18^ as well as antimicrobial potential^19–21^. Regarding its chemical profiles and biological activity, most research has been focused on EOs, in which α-pinene is usually the major constituent, followed by myrcene, sabinene, limonene, germacrene D, δ-cadinene and other terpenes and terpenoids. The chemical composition varies with seasonal factors, geographical origin, environmental conditions, and parts of the plant used for EO production (berries, needles)^16,22^. Having a typical Mediterranean distribution, *J. oxycedrus* grows from Turkey to Spain but it can also be found in the Atlas Mountains and in Iran^23^. The EOs of *J. oxycedrus* from Tunisia^24^ and Bulgaria^25^ demonstrated antimicrobial potential against Gram positive bacteria, including *Staph. aureus* and *Enterococcus faecalis*. Najar et al.^26^ confirmed these results and evidenced somehow an antimicrobial effect of the *Juniperus* EOs on *L. monocytogenes*. Cosentino et al.^22^ described the antimicrobial activity of Sardinian EOs of *J. communis* and *J. oxycedrus* and compared it to EOs from *J. turbinate*, founding that the latter was more active against fungi, particularly *Aspergillus flavus*.

Besides the EOs, non-volatile constituents of *Juniperus* sp. have been reported for biological activities in a small number of publications. These are mostly tested for antioxidant activities, however, the methanolic extracts of this genus were found to inhibit, to different extent, the growth of 57 strains belonging to 24 bacterial species, including methicillin-resistant *Staph. aureus* (MRSA) strains, *Enterobacter* spp., *Bacillus* spp.^21,23,27^.

Despite their great potential, the application of plant extracts in prolonging shelf life and assuring safety of foods is still limited. This is due to the variability of extract composition and conditions adopted in the antimicrobial activity tests, which affect their effects against target microorganisms, as well as to the absence of sufficient knowledge regarding the site and mode of action^28,29^. In addition, limitations can derive from their organoleptic and sensorial impact if used at inhibiting concentrations. Thus, many applications have been proposed in the framework of hurdle strategy, in which many sublethal factors are applied for controlling microbial growth. This philosophy is based on the principle that the accumulation of sublethal damages causes cell death or the inability to multiply^30^. On the other hand, the definition of dead cells in microbiology cannot be immediate. The traditional methods (plate counting) highlight the culturability of cells rather than their viability. Davey^31^ underlined the possible failure of these methods in detecting cryptobiotic, dormant, moribund, and latent cells. Indeed, these cells can have other measurable metabolic activities or be able to repair cellular integrity, thus restarting to grow during food storage.

This study aims to characterize and evaluate the antimicrobial effects of PEs and EOs obtained from *R. fruticosus* leaves and *J. oxycedrus* needles, harvested in the Mediterranean maquis of Croatia, against *L. monocytogenes* Scott A. The growth dynamics in the presence of sub-lethal concentrations of plant derivatives were modeled to highlight differences in the cell kinetics. Finally, *L. monocytogenes* cells cultivated in the presence of such plant derivates were analysed according to a flow cytometric protocol^32,33^, to better evidence their effect on cell viability and culturability.

## Results and discussion

### Characterization of plant derivatives

The major phenolic compounds of the *J. oxycedrus* needles and *R. fruticosus* leaves are listed in Table 1. The dominant phenolic acid in the *J. oxycedrus* needles extract was vanillic acid, with a concentration of 10.51 mg/l. Among flavonoids, the most abundant were apigenin and rutin, whose amounts were 7.66 and 6.95 mg/l, respectively. Regarding *R. fruticosus* leaves PE, the dominant phenolic acid was chlorogenic acid, while rutin was the most abundant flavonoid, with a concentration of 29.88 mg/l. Except for caffeic acid in *R. fruticosus* PE, the amounts of all other identified compounds were lower than 1 mg/l.

**Table 1:** Composition of the phenolic extracts (PEs) of *Juniperus oxycedrus* needles and *Rubus fruticosus* leaves obtained through HPLC analysis.

Phenolic compound	*J. oxycedrus*	*R. fruticosus*
Gallic acid	1.38 ± 0.01	0.13 ± 0.01
Caffeic acid	n.d.^a^	2.04 ± 0.04
Protocatechuic acid	0.32 ± 0.02	n.d
*p*-Hydroxybenzoic acid	0.81 ± 0.01	n.d
Vanillic acid	10.51 ± 0.16	n.d
Chlorogenic acid	n.d	6.22 ± 0.07
*p*-Coumaric acid	n.d	0.63 ± 0.01
(−)-Epicatechin	0.51 ± 0.01	0.11 ± 0.01
(+)-Catechin	4.86 ± 0.01	n.d
Rutin	6.95 ± 0.01	29.88 ± 0.39
Astringin	n.d	2.41 ± 0.02
Apigenin	7.66 ± 0.04	n.d
(−)Epigallocatechin gallate	0.72 ± 0.16	n.d
Total	33.72	41.42

Data are expressed as mg/l and are the means of three independent analyses.

^a^n.d.: not detected.

Previously, it has been reported that *J. oxycedrus* berry extracts have smaller content of total phenolics than the counterparts of the same genera^34^. Some authors recorded that the extraction by polar solvents, such as ethanol (used also in our study) increased the total phenolic yield during extraction. A similar output was reported by Orhan et al.^35^ for *J. oxycedrus* leaves ethanol extracts, however, the authors found that ethanolic extract of *J. oxycedrus* leaves had the highest amount of total phenols (206.19 ± 9.04 mg/g extract) among the five juniper species tested. There are only a few reports on the chemical compositions of the juniper leaf extracts. Mrid et al.^17^ identified salicylic acid (> 30 mg/1 g dw) and rutin (10.8 mg/g dw) as the most abundant compounds in *J. oxycedrus* needles methanolic extracts. Besides, the authors reported a high level of hesperidin (2.8 mg/g dw), and low concentrations of caffeic, *p*-coumaric and *p*-hydroxybenzoic acids (< 2 mg/g dw). About the chemical profile of the genera, Dziedzinski et al.^19^ found that the *J. communis* shoots were particularly rich in caffeic, ferulic, chlorogenic, and gallic acids (> 1000 µg/g dw), but had a small abundance of flavonoids (< 1 µg/g dw).

The blackberry fruits have been intensively studied and were found to be a rich source of biologically active phenolic compounds, particularly gallic acid and rutin^36–38^. On the other hand, a small number of publications indicate that the blackberry leaves can also be a good source of diverse groups of phenolic compounds. In general, blackberry leaves are rich in *p*-hydroxybenzoic and hydroxycinnamic acids (caffeic, gallic, ferulic, gentisic, vanillic, *p-*coumaric and others), ellagitannins, and flavonoids such as quercetin, mirycetin, luteolin, apigenin, kaempferol, catechin, and epicahechin^38,39^. Oszmiański et al.^39^ identified and quantified flavonoid derivatives of quercetin, kaempferol, luteolin and apigenin in different *Rubus* species in the range from 8.68 to 61.27 mg/g dry matter. They gave an overview of phenolic composition of 26 different wild blackberry leaves and identified thirty-three phenolic compounds, including 15 flavonoids (five kaempferol and 10 quercetin derivatives), nine phenolic acid derivatives, three derivatives of ellagic acid, and two flavones (apigenin and luteolin). The authors did not analyse *R. fruticosus* leaves. The content of quercetin and kaempferol in *R. fruticosus* leaves was determined by Gudej and Tomczyk^40^. Quercetin and kaempferol were found in ranges respectively of 0.16–0.31 and 0.11–0.15% of dry weight of the samples.

In Table 2 the characterization of the EO obtained from *J. oxycedrus* needles is reported. Almost all the molecules identified were terpenes or terpenoids, representing 88.11% of the total peak area. Table 2 reports only the molecules representing at least 0.5% of the total area (34 out 119 of volatiles identified). Limonene, α-pinene and manoyl oxide accounted for more than 30% of peak areas (13.59, 10.71 and 8.41%, respectively) while 3-carene, 4(15),5-muuroladiene and α-curcumene represented 4.12, 3.24 and 3.50% of the EO components. Minor amounts of δ-cadinene, β-pinene, and β-myrcene were also detected.

**Table 2:** Composition of essential oils (EOs) of *Juniperus oxycedrus* needles obtained through GC–MS analysis.

No	RT^a^	Compound	%
1	18.33	3-Carene	4.12 ± 0.09
2	19.12	α-Pinene	10.79 ± 0.19
3	21.38	β-Pinene	0.88 ± 0.02
4	22.08	β-Myrcene	0.94 ± 0.04
5	23.95	*o*-Cymene	0.61 ± 0.03
6	24.38	Limonene	13.59 ± 0.09
7	27.41	*p*-Menth-4(8)-ene	1.14 ± 0.01
8	27.89	Linalool	0.56 ± 0.01
9	29.37	Campholenic aldehyde	1.18 ± 0.05
10	30.12	(*E*)-pinocarveol	1.00 ± 0.01
11	30.42	Verbenol	1.68 ± 0.05
12	30.58	1.3-Cycloheptadiene	0.55 ± 0.01
13	31.47	1.3.5-Heptatriene	1.29 ± 0.03
14	32.15	*p*-Cymen-8-ol	1.06 ± 0.05
15	32.69	*p*-Menth-1-en-8-ol	0.65 ± 0.01
16	33.00	α-Thujenal	0.73 ± 0.06
17	33.25	Bornyl acetate	1.21 ± 0.01
18	33.67	(−)-Verbenone	1.11 ± 0.06
19	34.06	(*Z*)-Carveol	1.68 ± 0.03
20	35.27	(+)-Carvone	1.03 ± 0.01
21	42.84	α-Cedrene	1.94 ± 0.05
22	43.57	Caryophyllene	0.60 ± 0.01
23	44.01	4(15).5-Muuroladiene	3.24 ± 0.04
24	45.02	α-Caryophyllene	0.53 ± 0.01
25	46.00	α-Curcumene	3.50 ± 0.02
26	47.48	α-Amorphene	2.03 ± 0.07
27	47.79	δ-Cadinene	2.04 ± 0.03
28	48.53	α-Copaene-11-ol	0.55 ± 0.01
29	48.63	α-Calacorene	1.04 ± 0.06
30	50.31	Caryophyllene oxide	1.38 ± 0.01
31	51.00	Cedrol	0.84 ± 0.05
32	54.02	Farnesol	0.75 ± 0.01
33	60.15	Manoyl oxide	8.41 ± 0.06
34	61.30	ar-abietatriene	1.19 ± 0.01
		Total identified compounds	88.11

Data are expressed as relative percentages (± SD) of each peak area with respect to the total peak area and are the means of three independent analyses. Only peak with area higher than 0.5% are reported.

^a^Retention time (min).

The composition of *Juniperus* spp. EO reported in the literature is highly variable and depends on the species, the geographic area and the part of the plant used for the production. Α-pinene was the major compound in hydrodistilled *J. communis* berries EO from Portugal, followed by β-pinene and limonene^16^. The same authors also tested two commercial samples of the same EO in which α-pinene and β-pinene were again among the most important constituents, but their concentrations were markedly lower. Medini et al.^24^ studied the EOs obtained from needles of *J. oxycedrus* harvested in different Tunisian localities and found that the major constituents were α-pinene (which ranged from 16.0 to 49.6%), sabinene (0–12.1%), p-cimene (0–14.5%), germacrene D (0.5–9.0%) and manoyl oxide (2.5–6.4%). α-pinene was again the main terpene found in Italian *J. oxycedrus* needles EO ranging from 31.5 to 61.8%, followed by germacrene D^26^. A Bulgarian EO from *J. oxycedrus* leaves presented a composition similar to the EO of this study and, in addition to α-pinene and limonene, relevant amounts of manoyl oxide, caryophyllene oxide, abietatriene, curcumene, and β-caryophyllene^25^.

One hundred eighteen molecules (representing 90.94% of the total peak area) were identified in the EO obtained from *R. fruticosus* leaves. Table 3 reports the 36 compounds that accounted for a percentage higher than 0.5. The major components of the EO were monoterpenes such as geraniol (13.67%), β-citronellol (4.61%), linalool (4.13%), α-terpineol (3.05%) and citral (2.40%). Phytol, an acyclic diterpene alcohol, was present at a concentration of 4.87%, while β-ionone, typically giving a scent of violet and involved in vitamin A metabolism, accounted for 3.68% and olivetol for 3.02%. Several alkanes were also detected (tetradecane, hexadecane, eicosane, heneicosane), together with fatty acids (octanoic, decanoic, dodecanoic, tetradecanoic and hexadecenoic acid). Interestingly, also methyl salicylic acid was detected at a concentration of 1.25%.

**Table 3:** Composition of essential oils (EO) of *Rubus fruticosus* leaves obtained through GC–MS analysis.

No	RT^a^	Compound	%
1	27.92	Linalool	4.13 ± 0.33
2	31.49	1-Nonanol	0.59 ± 0.03
3	31.84	Octanoic acid	0.57 ± 0.32
4	32.69	α-Terpineol	3.05 ± 0.29
5	32.83	Methyl salicylate	1.25 ± 0.02
6	33.22	Decanal	1.04 ± 0.12
7	34.36	β-Citronellol	4.61 ± 0.15
8	35.01	Citral	2.40 ± 0.22
9	35.70	Geraniol	13.67 ± 1.09
10	37.82	Geranyl formate	0.50 ± 0.12
11	38.05	Undecanal	0.58 ± 0.09
12	40.75	*n*-Decanoic acid	1.04 ± 0.50
13	41.76	(*E*)-β-Damascenone	0.82 ± 0.13
14	42.20	Tetradecane	0.59 ± 0.13
15	44.49	Geranylacetone	0.63 ± 0.11
16	46.14	β-Ionone	3.68 ± 0.55
17	46.88	α-Farnesene	2.01 ± 0.24
18	47.41	3-Amino-2-cyclohexen-1-one	0.95 ± 0.04
19	47.70	Olivetol	3.02 ± 0.24
20	49.03	Dodecanoic acid	3.11 ± 1.66
21	49.10	Nerolidol	1.03 ± 0.59
22	50.32	Hexadecane	0.62 ± 0.09
23	51.03	Linalyl acetate	0.77 ± 0.11
24	53.76	Hexadecanal	3.27 ± 0.40
25	54.86	Tetradecanoic acid	0.81 ± 0.45
26	56.12	Tetradecanal	2.18 ± 0.25
27	56.70	Hexahydrofarnesyl acetone	1.33 ± 0.19
28	57.71	Eicosane	0.80 ± 0.14
29	58.11	16-Octadecenal	1.87 ± 0.28
30	58.17	Farnesyl acetone	0.65 ± 0.05
31	58.88	*n*-Hexadecanoic acid	1.17 ± 1.00
32	61.23	5-Octadecene	0.57 ± 0.03
33	61.47	Heneicosane	0.89 ± 0.25
34	61.90	Phytol	4.87 ± 0.79
35	62.49	Oleic acid	2.02 ± 1.02
36	64.45	Octadecanale	0.52 ± 0.03
		Total identified compounds	90.49

Data are expressed as relative percentages (± SD) of each peak area with respect to the total peak area and are the means of three independent analyses. Only peak with area higher than 0.5% are reported.

^a^Retention time (min).

The literature concerning the EO composition of *Rubus* spp. is scarce. In a study by Wajs-Bonikowska et al.^41^ EOs from *R. fruticosus* pomace obtained from hydrodistillation or using supercritical CO_2_ were compared. The main constituents were non-saturated aliphatic aldehydes. Among terpenes, α-copaene and p-cymene were the most relevant, but their concentration was rather low. Zhang et al.^42^ characterized the EO obtained from leaves of *R. pungens* which was composed of 36% of sesquiterpenes (including γ-elemene and β-caryophyllene). Another study demonstrated that the vegetative stage of the plant dramatically influenced the EO composition of *R. ulmifolius*. Anyway, the most relevant compounds detected were α-pinene, 1,8-cineole, linalool, geraniol, and, among aldehydes, (*E*)-2-hexenal and nonanal^43^.

### Effect of plant derivatives on *Listeria monocytogenes* growth kinetics

The PEs and the EOs of *R. fruticosus* and *J. oxycedrus* were firstly tested to define the MIC of the plant derivatives against *L. monocytogenes*. The results (data not shown) indicated that MIC was 1.5 mg/ml for the EO of *R. fruticosus* and 2 mg/ml for the EO of *J. oxycedrus* and for both the PEs. As already observed, the comparison between MIC of plant derivatives from different studies can be difficult because of the variable composition of derivatives and especially the lack of standardized test procedures^29,44^. In addition, literature is scarce on the MIC of the two species considered. In any case, a MIC of 2 mg/ml against *L. monocytogenes* has been reported for gallic acid^45^. Cosentino et al.^22^ found a minimum lethal concentration of J*. oxycedrus* EO at 900 mg/l for *L. monocytogenes* (and 250 mg/l for δ-carene) while lower MIC (approx. 32 mg/l) were observed by Najar et al.^26^, but in this latter case the time of incubation was 24 h, against the 48 of this study.

To better understand the effects of such plant derivatives on *L. monocytogenes*, the cells were inoculated in BHI medium and incubated at 20 °C containing sublethal doses (corresponding to 50% of MIC) of these PEs and EOs. The choice to use sublethal concentrations was aimed to assess the ability of cells to repair the damages induced by plant derivatives and the effects of the presence of these substance on the growth dynamics. The culturability was monitored over time by plate counting and compared with the control grown in the absence of inhibiting products. The distribution of the observations followed two distinct trends. The first, characterizing the control and the sample with PE of *R. fruticosus* leaves, represented a classical growth curve, with three distinct phases: lag, exponential and stationary phase. In this case, the experimental data were fitted with the classical Gompertz equation^46^. Conversely, the remaining samples showed, to a different extent, a first step in which cells decreased their culturability, after which the growth restarted with an exponential phase reaching the stationary phase and the maximum cell concentration. These experimental data were therefore fitted a double-peaked Gompertz model^47^, in which the first step describes the diminution of cell counting and the second the increase of cell number up to the reaching of the stationary phase.

**Figure 1:**
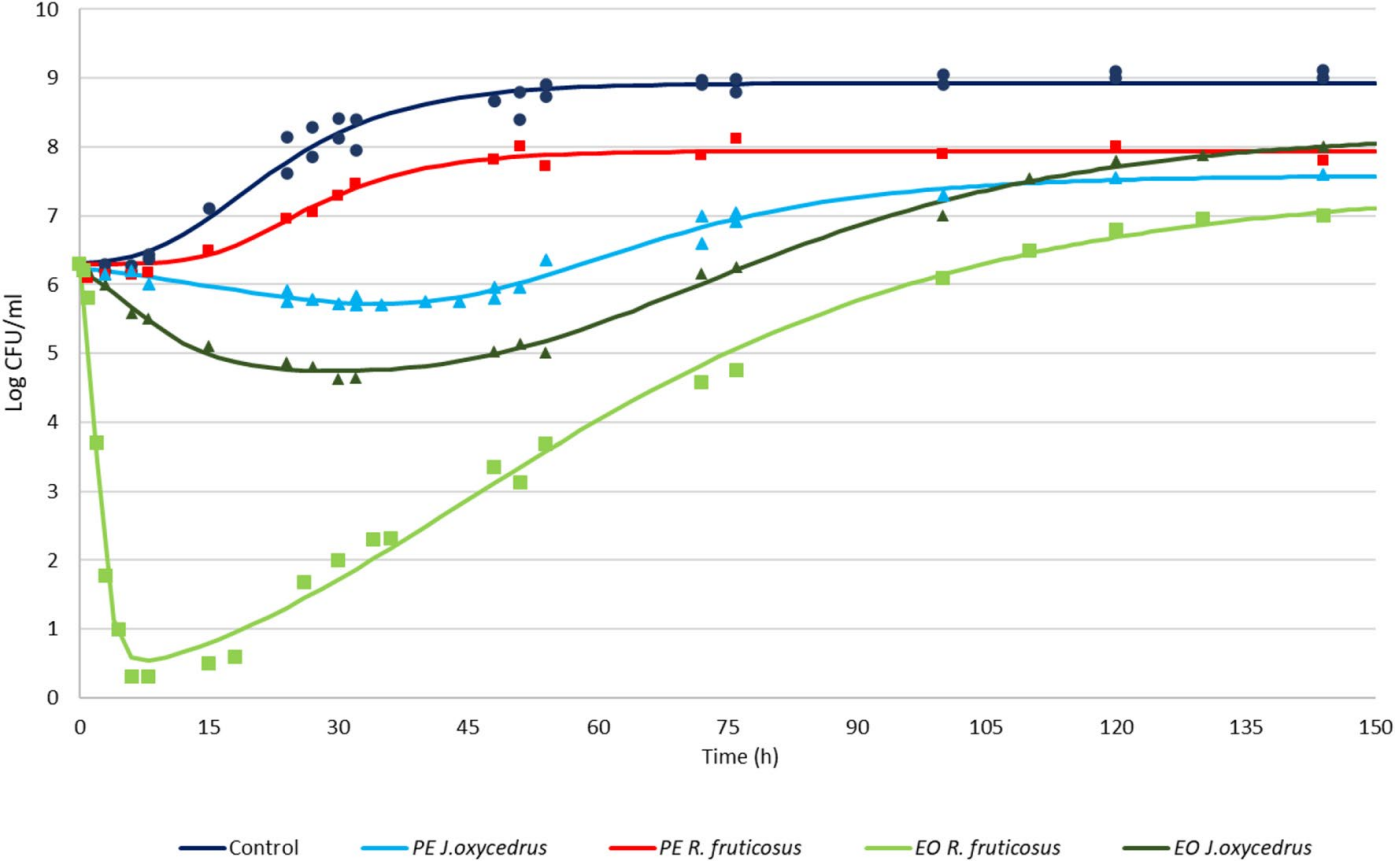
*L. monocytogenes* Scott A growth kinetics during incubation at 20 °C in the presence of different concentrations of plant derivatives: 1 mg/ml of phenolic extracts (PEs) of *J. oxycedrus* needles or *R. fruticosus* leaves, 1 mg/ml of essential oil (EO) of *J. oxycedrus* needles, 0.75 mg/l of EO of *R. fruticosus* leaves. The points represent the experimental data obtained by plate counting, while curves are the relative fitted models obtained with Gompertz equation.

The parameter estimates for the two models used are reported in Table 4, together with the maximum cell concentration attained according to the models and some diagnostics of fitting, while Fig. 1 reports the experimental points and the relative fitted models for each condition. The presence of the PE of *R. fruticosus* leaves determined a prolongation in the lag phase with respect to the control (14.28 h vs. 7.84 h), a slower exponential phase growth rate (0.066 (log CFU/ml)/h vs. 0.093 (log CFU/ml)/h) and a final concentration of 1 log unit lower than the control (7.94 (log CFU/ml)/h) vs. 8.92 (log CFU/ml)/h)). The PE of *J. oxycedrus* needles was more effective, and determined an initial decrease of the population culturability, even if low (0.788 log CFU/ml). Then, after 40.66 h, the cell number started to increase, although with a maximum growth rate (0.043 (log CFU/ml)/h)) lower with respect to the control and the sample containing *R. fruticosus* PE, and reached a maximum cell concentration of 7.58 log CFU/ml.

**Table 4:** *L. monocytogenes* Scott A growth parameters in the presence of different plant derivatives (phenolic extract, PE or essential oil, EO), estimated by modeling the data from plate counting (log CFU/ml) with the Gompertz equation.

Sample	k	A_1_^a^	µ_max1_^a^	λ_1_^a^	A_2_	µ_max2_	λ_2_	Max cell load	R	RMSE
Control	6.30	–	–	–	2.624	0.093	7.84	8.92	0.994	0.005
*J. oxycedrus* PE	6.30	− 0.788	− 0.020	− 1.11	2.070	0.043	40.66	7.58	0.982	0.007
*R. fruticosus* PE	6.30	–	–	–	1.639	0.066	14.28	7.94	0.988	0.007
*J. oxycedrus* EO	6.30	− 1.689	− 0.078	− 2.83	3.632	0.050	43.67	7.99	0.994	0.015
*R. fruticosus* EO	6.30	− 6.200	− 1.926	0.38	7.326	0.079	10.03	7.33	0.995	0.046

The maximum cell concentration attained according to the models and some diagnostics of fitting are also reported.

^**a**^Parameters estimated only for the double-peaked Gompertz model used when the initial cell concentration decreased before the growth started again.

The antimicrobial activity of PE from *Rubus* spp. has been already described. In particular, the anti-listerial effect of ethanolic extract from *R. discolor* was demonstrated in vitro and in yoghurt by Veličković et al.^11^, while the blackberry juice from *R. fruticosus* showed an inhibiting effect on *L. monocytogenes*, *Salmonella* Typhimurium and *E. coli* O157:H7 in milk and in BHI medium^15^.

The antimicrobial potential of molecules including also those found in the extracts studied in this work, such as gallic acid, vanillic acid, protocatecuic acid, rutin, apigenin, and caffeic acid, has been recently reviewed by Oulahal et al.^48^. For example, a MIC value of 2 mg/ml of gallic acid against *L. monocytogenes* is reported^45^. The role of phenolic compounds, used as vegetable extracts or pure molecules after isolation, in inhibiting this pathogen in model systems and foods has also been described^49^. The authors stated that, among the wide array of these compounds, some were able to exert protective effects against *L. monocytogenes*, and in particular stilbenes (resveratrol), cinnamic acids (cinnamul-3,4-dihydroxy-α-cyanocinnamate, caffeic acid 1,1-dimethylallyl ester), benzoic acids (butyl gallate, 3,4-dihydroxy-benzoic acid methyl ester), flavonoids (epigallocatechin gallate).

Concerning the mechanism of action, it has been recently demonstrated that phenolic extract from *Taraxacum officinale* containing rutin, caffeic acid, and chlorogenic acid caused in *Staph. aureus* membrane depolarization and permeabilization and altering the intracellular enzymatic activities^50^.

Quercetin and chlorogenic acid resulted effective against *L. monocytogenes* by involving redox imbalance which determined mortality increase^51^. At cytoplasmic level, apigenin can alter the activity of DNA gyrase and rutin interferes with topoisomerases, while catechins mainly perturb the membrane functionality^52^.

Both the EOs were more effective in inhibiting the growth of *L. monocytogenes*. In particular, the EO from *R. fruticosus* leaves determined a loss of culturabilty of almost the whole population (cell load reduction of about 6 log CFU/ml) in a few hours of incubation. However, after about 10 h multiplication of survivors began and determined a maximum estimated cell concentration of 7.33 log CFU/ml. The EO of *J. oxycedrus* needles had a lower impact on initial cell culturability. Nevertheless, it determined a reduction of the initial cell concentration of 1.69 log CFU/ml, after which cell number increased reaching a final value of 7.99 log CFU/ml.

The EO of *J. oxycedrus* needles contained many terpenes, many of which have a known antimicrobial effect. β-myrcene, linalool, citral, geraniol, 3-carene, and cymene showed antimicrobial activity against several microorganisms^53^. A *J. communis* EO rich in α-pinene (47.8%) used in a marinade was effective in reducing *L. monocytogenes* concentration in beef meat^54^, while another EO of the same species (containing 14.1% β-myrcene, 9.5% sabinene, 8.4% limonene, 5.4% α-amorphene) was able to avoid the proliferation of this pathogen in fermented sausages^55^. The *J. oxycedrus* obtained from EO leaves studied by Semerdjieva et al.^25^ showed good antimicrobial activity against *Staph. aureus*. The same inhibiting effect against *Staph. aureus* was observed also in the leaf essential oil from Tunisian *J. oxycedrus*, while the action was scarce against the Gram negative *E. coli* and *S. enterica*^24^.

As observed in Table 2, terpenes, and in particular limonene, α-pinene, manoyl oxide, and 3-carene, are the most important constituents of the *J. oxycedrus* EO (approx. 45% of the total). α-pinene and 3-carene were the main constituents of a *Cupressus sempervirens* EO which inhibited *Staph. aureus* by affecting the activity of the efflux pump^56^. In addition, several papers report the possible antimicrobial action of molecules present in *J. oxycedrus* EO^53,57^.

On the other hand, independently of the efficacy of the various oils reported in the literature, it is well known that the inhibitory or bactericidal activity depends on their composition and the interaction between the components which can bring to relevant synergistic effects^58,59^. In fact, components with scarce antimicrobial activity if considered alone, may result in relevant inhibition if used in combination, as observed for linalool and β-pinene on *Saccharomyces cerevisiae*^60^.

The composition of *R. fruticosus* EO was characterised by the presence of terpenoids such as linalool, α-terpineol, β-citronellol, and geraniol, whose antimicrobial activity has been demonstrated^61–64^. Among other constituents, the antimicrobial activity of phytol and short chain fatty acids (such as octanoic, decanoic, dodecanoic acid) are described^65,66^.

### Effect of plant derivatives on *Listeria monocytogenes* viability

**Figure 2:**
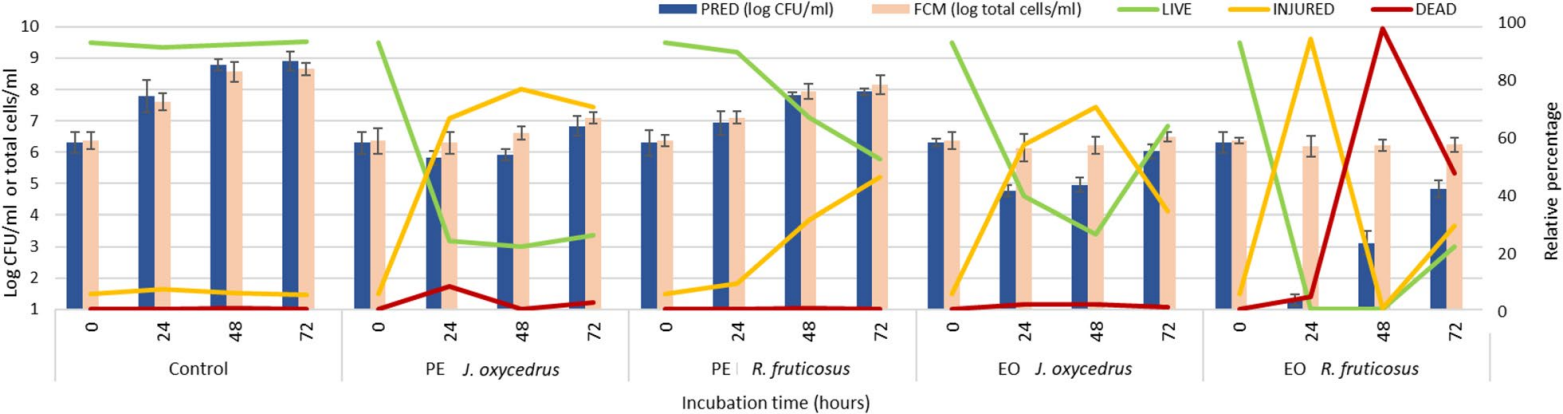
*L. monocytogenes* Scott A growth in the presence of different plant derivatives of *J. oxycedrus* and *R. fruticosus* (phenolic extract, PE or essential oil, EO) after 24, 48 and 72 h of incubation at 20 °C. The histograms represent the comparison between the data of cell culturability (expressed as log CFU/ml predicted by the models) and the total cells detected by FCM analysis (log total cells/ml). For these latter, also the relative percentages of live, injured and dead cells for each condition (as green, yellow and red lines, respectively) are reported.

With the aim to better investigate the impact of plant derivatives on *L. monocytogenes* physiological state, cells were analysed with a flow cytometric protocol to highlight the occurrence of different subpopulations characterized by different physiological states. Figure 2 summarized the results after 0, 24, 48, and 72 h. In particular, the data of cell culturability (expressed as log CFU/ml predicted by the models) were compared to the total cells detected by flow cytometry. For this latter, also the relative percentages of viable, injured and dead cells for each condition were reported.

Regarding the control, the data of plate counting and flow cytometric analysis were similar, without significant differences, and, in all cases, the viable cells represented about 94% of the total population. In the sample added with the PE of *J. oxycedrus* needles, the data of viability were coherent with the culturability predicted. This extract after 24 h mainly determined the presence of a high percentage of injured cells (67.65%), while live cells represented 24.26% of the total events. Interestingly, this ratio between alive and injured cells remained quite constant throughout the incubation time (72 h). The presence of such injured population can be responsible for the extended length of the lag phase (approx. 40 h vs. 8 h for the control), due to the need to overcome cell damage before starting multiplication. Indeed, similar behaviors were previously observed for *L. monocytogenes* after exposure to environmental stresses^67^.

The PE of R*. fruticosus* leaves, which caused a reduction of one log unit of the final maximum cell concentration, determined an increasing proportion of injured cells (from 9.09% after 24 h to 46.83% after 72 h). The different trend of cell injury between the two PEs is likely due to their different composition: indeed, in this case the occurrence of injury in the cell population did not result in a remarkable prolongation of lag phase (about 14 h), but the increasing ratio of injured cells overtime could be explained by the effect of *R. fruticosus* PE constituents, particularly rutin and chlorogenic acid, whose ability to affect cell membrane permeability (thus allowing higher retention of propidium iodide, used in this study to detect injured cells) has been demonstrated for some Gram positive bacteria^50^.

Concerning the EO of *J. oxycedrus* needles, the data of culturability after 24 and 48 h of incubation were lower if compared with the total cells detected by flow cytometry. This could be due to a decrease of cells recognized as live and the relative increase in the percentage of injured cells (58.24 and 71.59% after 24 and 48 h, respectively), being the latter likely responsible for the significant increase of lag phase (about 44 h), as previously observed for the *J. oxycedrus* needle PE. In addition, the number of total cells did not markedly change, indicating that the increase of culturable cells within the first 72 h of incubation depended on the recovery of injured cells (which passed from 71.59% at 48 h to 34.51% at 72 h) rather than on a multiplication. After 72 h an opposite trend was observed, suggesting an active response of the target strain to overcome the stress induced by the presence of such plant derivatives and to start to multiply, as stated by the growth kinetics (Fig. 1).

The EO of *R. fruticosus* leaves showed the more evident effects. Besides, in this case, the number of total cells detected through flow cytometry did not significantly change during the 72 h incubation. The predicted culturability at 24 h was 1.31 log CFU/ml, while the viability was higher, i.e., 2.88 log cell/ml, which corresponded to 0.05% of cells recognized as live in the total population. Under this condition, the major part of cells was injured (95.65%). More than 99% of cells were classified as dead and 0.1% as alive at 48 h. After 72 h the data of culturability increased, and also the ratio of live cells, that reached 20.02% of the total population. Based on these results, we hypothesized that EO of *R. fruticosus* was the only plant derivative able to exert a bactericidal effect, i.e., to induce a relevant increase of dead cells. Indeed, the initial discrepancy between viability and culturability could suggest the occurrence of viable but not culturable cells (VBNC), a great safety concern because of the ability to reverse to a viable status^33^. However, considering both plate counting and flow cytometry analysis, it seems that the increase of culturability (log CFU/ml) and viability (i.e., % of live cells) observed starting from 48 h of incubation was likely due to the multiplication of the few cells survived to the effect of this EO rather than a resuscitation phenomenon.

Conversely, the other plant derivatives tested were able to sub-lethally damage the *L. monocytogenes* cells, determining different extents of cell injury that indicate differences in the mechanism of antimicrobial action, particularly in terms of cell membrane integrity. This confirmed the suitability of flow cytometry and fluorescent staining procedures in the study of cell injury caused by stress factors to better elucidate the different patterns of metabolic responses and the potential safety risks^67^.

## Conclusions

The plant derivatives from *J. oxycedrus* needles and *R. fruticosus* leaves considered in this study were able to slow down the growth kinetics of *L. monocytogenes* (monitored by plate counting), even if added at concentrations corresponding to half of MIC. In general, both PEs and EOs significantly reduced the final maximum cell culturability of approx. 1 log unit. PEs were less effective than EOs in limiting the growth performance of *L. monocytogenes.* Indeed, no loss of culturability was observed for the PE of *R. fruticosus* leaves and only a weak decline was observed for *J. oxycedrus* needles PE. The antilisterial activity was more relevant using EOs, particularly the one derived from *R. fruticosus* leaves. This latter caused the most severe effects, since it was able, in a few hours of incubation, to almost completely inhibit the cell culturability. According to the flow cytometry analysis, the presence of this EO was able to induce the death of the major part of cells (> 99%). Concerning the other plant derivatives, the discrepancy observed in some cases between viability and culturability could indicate the presence of cells not able to grow in culture media (at least in the adopted conditions) whose fate needs to be further investigated for a deeper comprehension of their possibility to revert to culturable status, contributing to an overestimation of the effect of these antimicrobial substances.

The results of this study increase the knowledge of these underused raw materials such as blackberry leaves and juniper needles, that can be exploited in food production and other industrial sectors.

## Materials and methods

### Bacterial strain and growth condition

The strain used in this study was *Listeria monocytogenes* Scott A belonging to the collection of the Department of Agricultural and Food Sciences (University of Bologna). The strain was maintained in BHI medium (Oxoid, Basingstoke, UK) with 30% (w/v) glycerol at − 80 °C and, before the experiments, pre-cultivated twice (37 °C for 24 h) in BHI medium.

### Plant collection and preparation of phenolic extracts (PEs) and essential oils (EOs)

The *R. fruticosus* leaves and *J. oxycedrus* needles were harvested in August of 2020 at the mountain Kozjak, Croatia (43°58′15″N, 16°32′39″E) at the altitude of 420 m. The plant material was dried in a shady place at room temperature for 7 days. To obtain the PEs, dried materials were extracted in 50% EtOH using the MAE (advanced microwave extraction system ETHOS X, Milestone Srl, Sorisole, Italy, 600 W, 5 min) method. After the extraction the EtOH was evaporated and the extracts freeze-dried and stored in a cool dark place until analyses^68^. Isolation of EOs was done by hydrodistillation. Briefly, about 100 g of the dried material was immersed in a flask with distilled water in Clevenger-type apparatus for 3 h. Pentane and diethyl ether (1:1, v/v) were used for trapping the volatile compounds. The obtained essential oil was dried over anhydrous sodium sulphate and the EOs stored at 4 °C in dark vials until analyses^69^.

### Characterization of PEs through HPLC analysis

Freeze-dried extracts were dissolved in 50% ethanol (10 mg/10 ml) and the individual phenolics of PEs were identified and quantified using an HPLC Ultimate 3000 (Thermo Fisher Scientific, Wathman, MA, SAD) equipped with a UV–Vis DAD using the method of Generalić et al.^70^ with some modifications. The separation was carried out using a Syncronis™ C18 Column (250 × 4.6 mm, 5 µm particle size, Thermo Fisher Scientific, Waltham, MA, USA). The column temperature was set at 25 °C, the volume of the injected sample was 10 µl and the flow rate was 0.8 ml/min. The total runtime of the method was 80 min with the following conditions: a gradient consisting of solvent A (water/formic acid, 98:2, v/v), solvent B (acetonitrile), and solvent C (methanol) applied as follows: from 96% A, 2% B, 2% C at 0 min to 50% A, 25% B, 25% C at 40 min, to 40% A, 30% B, 30% C at 45 min, to 0% A, 50% B, 50% C at 60 min, to 96% A, 2% B, 2% C at 70 min, maintaining 96% A, 2% B, 2% C for 10 min (80 min). The peaks of individual phenolics were identified by comparing their retention times and absorption spectra (at two wavelengths 280 and 320 nm) with those acquired for corresponding standards. The identified compounds were quantified using external standard calibration curves (injected in five different concentrations). Data were reported as means of the two independent analyses. The results were expressed in mg of compound per l of extract (mg/l). All used reagents, solvents, and standards were purchased from Sigma (Sigma–Aldrich GmbH, Steinheim, Germany), Merck (Darmstadt, Germany), Fluka (Buch, Switzerland), and Kemika (Zagreb, Croatia) and were of adequate analytical grade.

### Characterization of EOs through GC–MS analysis

The composition of EOs obtained from *R. fruticosus* leaves and *J. oxycedrus* needles was analyzed using a GC–MS (Shimadzu QP2010, Shimadzu, Kyoto, JP) equipped with an autosampler and a DB-5 60 m × 0.25 mm × 0.25 μm column (Agilent Technologies Italia Spa, Milano, Italy), following a protocol previously reported^69^. The EOs were resuspended in hexane and 1 µl was injected in the following gas chromatographic conditions: injection temperature 260 °C; interface temperature 280 °C; ion source 220 °C; carrier gas (He) flow rate 30 cm/s; splitting ratio 1:20. The oven temperature was programmed as follows: 40 °C for 4 min; from 40 to 175 °C with a 3 °C/min rate of increase; from 175 to 300 °C with a 7 °C/min increase, then holding for 10 min. The compounds were identified by comparing their spectra with those reported in NIST 8.0 library (US National Institute of Standards and Technology). For each sample, the EOs composition was expressed as a relative percentage of each single peak area with respect to the total peak area. Data reported are the means of three repetitions. Only the compounds whose peak area was higher than 0.5% of the total peak area are reported in the final tables.

### Determination of minimum inhibiting concentration (MIC)

The in vitro antimicrobial activity of these plant derivatives against the target strain was assessed with broth microdilution method using microtiter plates (Corning Incorporated, N.Y., U.S.A.) as previously reported^32^. For the determination of cell growth/no growth, 198 μl of BHI broth inoculated with *L. monocytogenes* (about 6 log CFU/ml) were placed into 200 μl microtiter wells. Plant derivatives were dissolved in ethanol and 2 μl of these solutions were added to each well to obtain final concentrations ranging between 0 and 3 mg/ml. Microtiter plates were incubated at 37 °C for 48 h. The MIC was defined as the lowest concentration of plant derivatives able to prevent a visible growth in the well.

### Effect of plant derivatives on *Listeria monocytogenes* growth

To assess the effect of sub-lethal concentrations of plant derivates on *L. monocytogenes* Scott A growth, the target strain was inoculated in BHI broth (about 6 log CFU/ml), in the presence of amounts of such extracts corresponding to 50% of MIC, according to a protocol previously adopted in our studies^32^. In particular, 1 mg/ml of plant derivates was added in each condition, with the exception of *R. fruticosus* EO, whose concentration was 0.75 mg/ml. The samples were then incubated at 20 °C and monitored for 144 h. At defined times, samples were collected to assess culturability (by plate counting) and viability (by flow cytometry). For plate counting, appropriate decimal dilutions were plated onto BHI agar medium and incubated at 37 °C for 48 h.

### Growth/deactivation kinetics modelling

The data obtained from plate counting were modeled with the Gompertz equation as modified by Zwietering et al.^46^,

$$y = k + A \cdot e^{{ - e^{{\left[ {\left( {\frac{{\mu_{max} \cdot e}}{A}} \right) \cdot \left( {\lambda - t} \right) + 1} \right]}} }}$$

where *y* is the cellular load at time t, *k* is a constant representing the initial cellular load, the parameter *A* represents the difference between maximum cellular load reached and the initial cell concentration, the parameter *µ*_*max*_ is the maximum log CFU/ml increase rate in exponential phase and the parameter *λ* is the lag phase duration.

Since in some samples an initial decrease of cell counts was observed, a double-peaked Gompertz equation was used to fit also the first part of the curves^45^. In this case the model was:

$$y = k + \left( {A_{1} \cdot e^{{ - e^{{\left[ {\left( {\frac{{\mu_{max1} \cdot e}}{{A_{1} }}} \right) \cdot \left( {\lambda_{1} - t} \right) + 1} \right]}} }} } \right) + \left( {A_{2} \cdot e^{{ - e^{{\left[ {\left( {\frac{{\mu_{max2} \cdot e}}{{A_{2} }}} \right) \cdot \left( {\lambda_{2} - t} \right) + 1} \right]}} }} } \right)$$

where *y* is the cellular load at time t, *k* is a constant representing the initial cellular load, the parameter *A*_*1*_ represents the difference between the initial inoculum and the cellular load reached in the first phase of decrease, *A*_*2*_ represent the difference between the minimum cellular load after the first phase of decrease and the maximum cellular load reached in the second growth phase, *µ*_*max1*_ and *µ*_*max2*_ are the maximum log CFU/ml decrease or increase rate in exponential phase, respectively, and the parameters *λ*_*1*_ and *λ*_*2*_ are the lag phase duration of the two phases. In both cases, *k* was maintained constant at 6.30 log CFU/ml as determined by plate counting immediately after the inoculum.

Data modeling was performed using STATISTICA software (Statsoft Italia, Vigonza, Italy).

### Flow cytometry (FCM) analysis

Cell suspensions collected after 24, 48, and 72 h of incubation in the presence of the different plant derivatives were analysed with a flow cytometer Accuri C6 (BD Biosciences, Milan, Italy), using setting parameters, emission filters, and thresholds according to Arioli et al.^32^. Before analyses, samples were diluted (if needed) in filtered PBS and the cells were stained with SYBR-Green I (1×) and propidium iodide (PI) 7.5 µM at 37 °C for 15 min, in order to let the dye react with the cell. This dual staining allowed to distinguish three sub-populations corresponding to different physiological states: live, injured, and dead cells. The data obtained were analysed using the BD ACCURITM C6 software version 1.0 (BD Biosciences, Milan, Italy).

## Additional information

### Plants used in the study

Voucher specimens are deposited in herbarium of the laboratory of Department of Marine Studies, University of Split (Croatia), with the numbers UniST 52,120 (*Juniperus oxycedrus*) and UniSt 82,020 (*Rubus fruticosus)*. Plants were identified by prof. Frane Strikić, agronomist from the same University, and are available on reasonable request. No permission was needed for their collection because these are wild non-protected plants (no risk of extinction). Experimental research and field studies on plants are compliant with relevant institutional, national, and international guidelines and legislation.

## Data Availability

The datasets used and/or analysed during the current study available from the corresponding author on reasonable request.
